# The JASP guidelines for conducting and reporting a Bayesian analysis

**DOI:** 10.3758/s13423-020-01798-5

**Published:** 2020-10-09

**Authors:** Johnny van Doorn, Don van den Bergh, Udo Böhm, Fabian Dablander, Koen Derks, Tim Draws, Alexander Etz, Nathan J. Evans, Quentin F. Gronau, Julia M. Haaf, Max Hinne, Šimon Kucharský, Alexander Ly, Maarten Marsman, Dora Matzke, Akash R. Komarlu Narendra Gupta, Alexandra Sarafoglou, Angelika Stefan, Jan G. Voelkel, Eric-Jan Wagenmakers

**Affiliations:** 1 University of Amsterdam, Amsterdam, Netherlands; 2 Nyenrode Business University, Breukelen, Netherlands; 3 University of California, Irvine, California USA; 4 Centrum Wiskunde & Informatica, Amsterdam, Netherlands; 5 Stanford University, Stanford, California USA

**Keywords:** Bayesian inference, Scientific reporting, Statistical software

## Abstract

Despite the increasing popularity of Bayesian inference in empirical research, few practical guidelines provide detailed recommendations for how to apply Bayesian procedures and interpret the results. Here we offer specific guidelines for four different stages of Bayesian statistical reasoning in a research setting: *planning* the analysis, *executing* the analysis, *interpreting* the results, and *reporting* the results. The guidelines for each stage are illustrated with a running example. Although the guidelines are geared towards analyses performed with the open-source statistical software JASP, most guidelines extend to Bayesian inference in general.

In recent years, Bayesian inference has become increasingly popular, both in statistical science and in applied fields such as psychology, biology, and econometrics (e.g., Andrews & Baguley, 2013; Vandekerckhove, Rouder, & Kruschke, 2018). For the pragmatic researcher, the adoption of the Bayesian framework brings several advantages over the standard framework of frequentist null-hypothesis significance testing (NHST), including (1) the ability to obtain evidence in favor of the null hypothesis and discriminate between “absence of evidence” and “evidence of absence” (Dienes, 2014; Keysers, Gazzola, & Wagenmakers, 2020); (2) the ability to take into account prior knowledge to construct a more informative test (Gronau, Ly, & Wagenmakers, 2020; Lee & Vanpaemel, 2018); and (3) the ability to monitor the evidence as the data accumulate (Rouder, 2014). However, the relative novelty of conducting Bayesian analyses in applied fields means that there are no detailed reporting standards, and this in turn may frustrate the broader adoption and proper interpretation of the Bayesian framework.

Several recent statistical guidelines include information on Bayesian inference, but these guidelines are either minimalist (Appelbaum et al., 2018; The BaSiS group, 2001), focus only on relatively complex statistical tests (Depaoli & Schoot, 2017), are too specific to a certain field (Spiegelhalter, Myles, Jones, & Abrams, 2000; Sung et al., 2005), or do not cover the full inferential process (Jarosz & Wiley, 2014). The current article aims to provide a general overview of the different stages of the Bayesian reasoning process in a research setting. Specifically, we focus on guidelines for analyses conducted in JASP (JASP Team, 2019; jasp-stats.org), although these guidelines can be generalized to other software packages for Bayesian inference. JASP is an open-source statistical software program with a graphical user interface that features both Bayesian and frequentist versions of common tools such as the *t* test, the ANOVA, and regression analysis (e.g., Marsman & Wagenmakers, 2017; Wagenmakers et al., 2018).

We discuss four stages of analysis: planning, executing, interpreting, and reporting. These stages and their individual components are summarized in Table 1. In order to provide a concrete illustration of the guidelines for each of the four stages, each section features a data set reported by Frisby and Clatworthy (1975). This data set concerns the time it took two groups of participants to see a figure hidden in a stereogram—one group received advance visual information about the scene (i.e., the VV condition), whereas the other group did not (i.e., the NV condition).^1^ Three additional examples (mixed ANOVA, correlation analysis, and a *t* test with an informed prior) are provided in an online appendix at https://osf.io/nw49j/. Throughout the paper, we present three boxes that provide additional technical discussion. These boxes, while not strictly necessary, may prove useful to readers interested in greater detail.

**Table 1:** A summary of the guidelines for the different stages of a Bayesian analysis, with a focus on analyses conducted in JASP.

Stage	Recommendation
Planning	Write the methods section in advance of data collection
	Distinguish between exploratory and confirmatory research
	Specify the goal; estimation, testing, or both
	If the goal is testing, decide on one-sided or two-sided procedure
	Choose a statistical model
	Determine which model checks will need to be performed
	Specify which steps can be taken to deal with possible model violations
	Choose a prior distribution
	Consider how to assess the impact of prior choices on the inferences
	Specify the sampling plan
	Consider a Bayes factor design analysis
	Preregister the analysis plan for increased transparency
	Consider specifying a multiverse analysis
Executing	Check the quality of the data (e.g., assumption checks)
	Annotate the JASP output
Interpreting	Beware of the common pitfalls
	Use the correct interpretation of Bayes factor and credible interval
	When in doubt, ask for advice (e.g., on the JASP forum)
Reporting	Mention the goal of the analysis
	Include a plot of the prior and posterior distribution, if available
	If testing, report the Bayes factor, including its subscripts
	If estimating, report the posterior median and *x%* credible interval
	Include which prior settings were used
	Justify the prior settings (particularly for informed priors in a testing scenario)
	Discuss the robustness of the result
	If relevant, report the results from both estimation and hypothesis testing
	Refer to the statistical literature for details about the analyses used
	Consider a sequential analysis
	Report the results of any multiverse analyses, if conducted
	Make the .jasp file and data available online

Note that the stages have a predetermined order, but the individual recommendations can be rearranged where necessary

## Stage 1: Planning the analysis

### Specifying the goal of the analysis.

We recommend that researchers carefully consider their goal, that is, the research question that they wish to answer, prior to the study (Jeffreys, 1939). When the goal is to ascertain the presence or absence of an effect, we recommend a Bayes factor hypothesis test (see Box 1). The Bayes factor compares the predictive performance of two hypotheses. This underscores an important point: in the Bayes factor testing framework, hypotheses cannot be evaluated until they are embedded in fully specified models with a prior distribution and likelihood (i.e., in such a way that they make quantitative predictions about the data). Thus, when we refer to the predictive performance of a hypothesis, we implicitly refer to the accuracy of the predictions made by the model that encompasses the hypothesis (Etz, Haaf, Rouder, & Vandekerckhove, 2018).

When the goal is to determine the size of the effect, under the assumption that it is present, we recommend to plot the posterior distribution or summarize it by a credible interval (see Box 2). Testing and estimation are not mutually exclusive and may be used in sequence; for instance, one may first use a test to ascertain that the effect exists, and then continue to estimate the size of the effect.

### Box 1. Hypothesis testing

The principled approach to Bayesian hypothesis testing is by means of the Bayes factor (e.g., Etz & Wagenmakers, 2017; Jeffreys, 1939; Ly, Verhagen, & Wagenmakers, 2016; Wrinch & Jeffreys, 1921). The Bayes factor quantifies the relative predictive performance of two rival hypotheses, and it is the degree to which the data demand a change in beliefs concerning the hypotheses’ relative plausibility (see Equation 1). Specifically, the first term in Equation 1 corresponds to the prior odds, that is, the relative plausibility of the rival hypotheses before seeing the data. The second term, the Bayes factor, indicates the evidence provided by the data. The third term, the posterior odds, indicates the relative plausibility of the rival hypotheses after having seen the data.

1 $$ \begin{array}{@{}rcl@{}} \underbrace{ \frac{p(\mathcal{H}_{1})}{p(\mathcal{H}_{0})}}_{\text{Prior odds}}   \times   \underbrace{ \frac{p(D \mid \mathcal{H}_{1})}{p(D \mid \mathcal{H}_{0})}}_{\text{Bayes factor}_{10}}   =   \underbrace{ \frac{p(\mathcal{H}_{1} \mid D)}{p(\mathcal{H}_{0} \mid D)}}_{\text{Posterior odds}} \end{array} $$

The subscript in the Bayes factor notation indicates which hypothesis is supported by the data. BF_10_ indicates the Bayes factor in favor of ${\mathscr{H}}_{1}$ over ${\mathscr{H}}_{0}$, whereas BF_01_ indicates the Bayes factor in favor of ${\mathscr{H}}_{0}$ over ${\mathscr{H}}_{1}$. Specifically, BF_10_ = 1/BF_01_. Larger values of BF_10_ indicate more support for ${\mathscr{H}}_{1}$. Bayes factors range from 0 to $\infty $, and a Bayes factor of 1 indicates that both hypotheses predicted the data equally well. This principle is further illustrated in Figure 4.

### Box 2. Parameter estimation

For Bayesian parameter estimation, interest centers on the posterior distribution of the model parameters. The posterior distribution reflects the relative plausibility of the parameter values after prior knowledge has been updated by means of the data. Specifically, we start the estimation procedure by assigning the model parameters a prior distribution that reflects the relative plausibility of each parameter value before seeing the data. The information in the data is then used to update the prior distribution to the posterior distribution. Parameter values that predicted the data relatively well receive a boost in plausibility, whereas parameter values that predicted the data relatively poorly suffer a decline (Wagenmakers, Morey, & Lee, 2016). Equation 2 illustrates this principle. The first term indicates the prior beliefs about the values of parameter *𝜃*. The second term is the updating factor: for each value of *𝜃*, the quality of its prediction is compared to the average quality of the predictions over all values of *𝜃*. The third term indicates the posterior beliefs about *𝜃*.

2 $$ \begin{array}{@{}rcl@{}} \underbrace{ p(\theta)}_{\underset{\text{about $\theta$}}{\text{Prior belief}} }   \times \overbrace{\underbrace{\frac{p(\text{data} \mid \theta)}{p(\text{data})}}}^{\underset{\text{ of specific } \theta}{\text{Predictive adequacy}} }_{\underset{\text{adequacy across all } {\theta}'s} {\text{Average predictive }}}   =      \underbrace{ p(\theta \mid \text{data})}_{\underset{\text{about $\theta$}}{\text{Posterior belief}} }. \end{array} $$

The posterior distribution can be plotted or summarized by an *x%* credible interval. An *x%* credible interval contains *x%* of the posterior mass. Two popular ways of creating a credible interval are the highest density credible interval, which is the narrowest interval containing the specified mass, and the central credible interval, which is created by cutting off $\frac {100-x}{2}\%$ from each of the tails of the posterior distribution.

### Specifying the statistical model.

The functional form of the model (i.e., the likelihood; Etz, 2018) is guided by the nature of the data and the research question. For instance, if interest centers on the association between two variables, one may specify a bivariate normal model in order to conduct inference on Pearson’s correlation parameter *ρ*. The statistical model also determines which assumptions ought to be satisfied by the data. For instance, the statistical model might assume the dependent variable to be normally distributed. Violations of assumptions may be addressed at different points in the analysis, such as the data preprocessing steps discussed below, or by planning to conduct robust inferential procedures as a contingency plan.

The next step in model specification is to determine the sidedness of the procedure. For hypothesis testing, this means deciding whether the procedure is one-sided (i.e., the alternative hypothesis dictates a specific direction of the population effect) or two-sided (i.e., the alternative hypothesis dictates that the effect can be either positive or negative). The choice of one-sided versus two-sided depends on the research question at hand and this choice should be theoretically justified prior to the study. For hypothesis testing it is usually the case that the alternative hypothesis posits a specific direction. In Bayesian hypothesis testing, a one-sided hypothesis yields a more diagnostic test than a two-sided alternative (e.g., Jeffreys, 1961; Wetzels, Raaijmakers, Jakab, & Wagenmakers, 2009, p.283).^2^

For parameter estimation, we recommend to always use the two-sided model instead of the one-sided model: when a positive one-sided model is specified but the observed effect turns out to be negative, all of the posterior mass will nevertheless remain on the positive values, falsely suggesting the presence of a small positive effect.

The next step in model specification concerns the type and spread of the prior distribution, including its justification. For the most common statistical models (e.g., correlations, *t* tests, and ANOVA), certain “default” prior distributions are available that can be used in cases where prior knowledge is absent, vague, or difficult to elicit (for more information, see Ly et al.,, 2016). These priors are default options in JASP. In cases where prior information is present, different “informed” prior distributions may be specified. However, the more the informed priors deviate from the default priors, the stronger becomes the need for a justification (see the informed *t* test example in the online appendix at https://osf.io/ybszx/). Additionally, the robustness of the result to different prior distributions can be explored and included in the report. This is an important type of robustness check because the choice of prior can sometimes impact our inferences, such as in experiments with small sample sizes or missing data. In JASP, Bayes factor robustness plots show the Bayes factor for a wide range of prior distributions, allowing researchers to quickly examine the extent to which their conclusions depend on their prior specification. An example of such a plot is given later in Figure 7.

### Specifying data preprocessing steps.

Dependent on the goal of the analysis and the statistical model, different data preprocessing steps might be taken. For instance, if the statistical model assumes normally distributed data, a transformation to normality (e.g., the logarithmic transformation) might be considered (e.g., Draper & Cox, 1969). Other points to consider at this stage are when and how outliers may be identified and accounted for, which variables are to be analyzed, and whether further transformation or combination of data are necessary. These decisions can be somewhat arbitrary, and yet may exert a large influence on the results (Wicherts et al., 2016). In order to assess the degree to which the conclusions are robust to arbitrary modeling decisions, it is advisable to conduct a multiverse analysis (Steegen, Tuerlinckx, Gelman, & Vanpaemel, 2016). Preferably, the multiverse analysis is specified at study onset. A multiverse analysis can easily be conducted in JASP, but doing so is not the goal of the current paper.

### Specifying the sampling plan.

As may be expected from a framework for the continual updating of knowledge, Bayesian inference allows researchers to monitor evidence as the data come in, and stop whenever they like, for any reason whatsoever. Thus, strictly speaking there is no Bayesian need to pre-specify sample size at all (e.g., Berger & Wolpert, 1988). Nevertheless, Bayesians are free to specify a sampling plan if they so desire; for instance, one may commit to stop data collection as soon as BF_10_ ≥ 10 or BF_01_ ≥ 10. This approach can also be combined with a maximum sample size (*N*), where data collection stops when either the maximum *N* or the desired Bayes factor is obtained, whichever comes first (for examples see ; Matzke et al., 2015;Wagenmakers et al., 2015).

In order to examine what sampling plans are feasible, researchers can conduct a *Bayes factor design analysis* (Schönbrodt & Wagenmakers, 2018; Stefan, Gronau, Schönbrodt, & Wagenmakers, 2019), a method that shows the predicted outcomes for different designs and sampling plans. Of course, when the study is observational and the data are available ‘en bloc’, the sampling plan becomes irrelevant in the planning stage.

### Stereogram example

First, we consider the research goal, which was to determine if participants who receive advance visual information exhibit a shorter fuse time (Frisby & Clatworthy, 1975). A Bayes factor hypothesis test can be used to quantify the evidence that the data provide for and against the hypothesis that an effect is present. Should this test reveal support in favor of the presence of the effect, then we have grounds for a follow-up analysis in which the size of the effect is estimated.

Second, we specify the statistical model. The study focus is on the difference in performance between two between-subjects conditions, suggesting a two-sample *t* test on the fuse times is appropriate. The main measure of the study is a reaction time variable, which can for various reasons be non-normally distributed (Lo & Andrews, 2015; but see Schramm & Rouder, 2019). If our data show signs of non-normality we will conduct two alternatives: a *t* test on the log-transformed fuse time data and a non-parametric *t* test (i.e., the Mann–Whitney *U* test), which is robust to non-normality and unaffected by the log-transformation of the fuse times.

For hypothesis testing, we compare the null hypothesis (i.e., advance visual information has no effect on fuse times) to a one-sided alternative hypothesis (i.e., advance visual information *shortens* the fuse times), in line with the directional nature of the original research question. The rival hypotheses are thus ${\mathscr{H}}_{0}: \delta = 0$ and ${\mathscr{H}}_{+}: \delta > 0$, where *δ* is the standardized effect size (i.e., the population version of Cohen’s *d*), ${\mathscr{H}}_{0}$ denotes the null hypothesis, and ${\mathscr{H}}_{+}$ denotes the one-sided alternative hypothesis (note the ‘+’ in the subscript). For parameter estimation (under the assumption that the effect exists), we use the two-sided *t* test model and plot the posterior distribution of *δ*. This distribution can also be summarized by a 95*%* central credible interval.

We complete the model specification by assigning prior distributions to the model parameters. Since we have only little prior knowledge about the topic, we select a default prior option for the two-sample *t* test, that is, a Cauchy distribution^3^ with spread *r* set to ${1}/{\sqrt {2}}$. Since we specified a one-sided alternative hypothesis, the prior distribution is truncated at zero, such that only positive effect size values are allowed. The robustness of the Bayes factor to this prior specification can be easily assessed in JASP by means of a Bayes factor robustness plot.

Since the data are already available, we do not have to specify a sampling plan. The original data set has a total sample size of 103, from which 25 participants were eliminated due to failing an initial stereo-acuity test, leaving 78 participants (43 in the NV condition and 35 in the VV condition). The data are available online at https://osf.io/5vjyt/.

## Stage 2: Executing the analysis

Before executing the primary analysis and interpreting the outcome, it is important to confirm that the intended analyses are appropriate and the models are not grossly misspecified for the data at hand. In other words, it is strongly recommended to examine the validity of the model assumptions (e.g., normally distributed residuals or equal variances across groups). Such assumptions may be checked by plotting the data, inspecting summary statistics, or conducting formal assumption tests (but see Tijmstra, 2018).

**Fig. 1:**
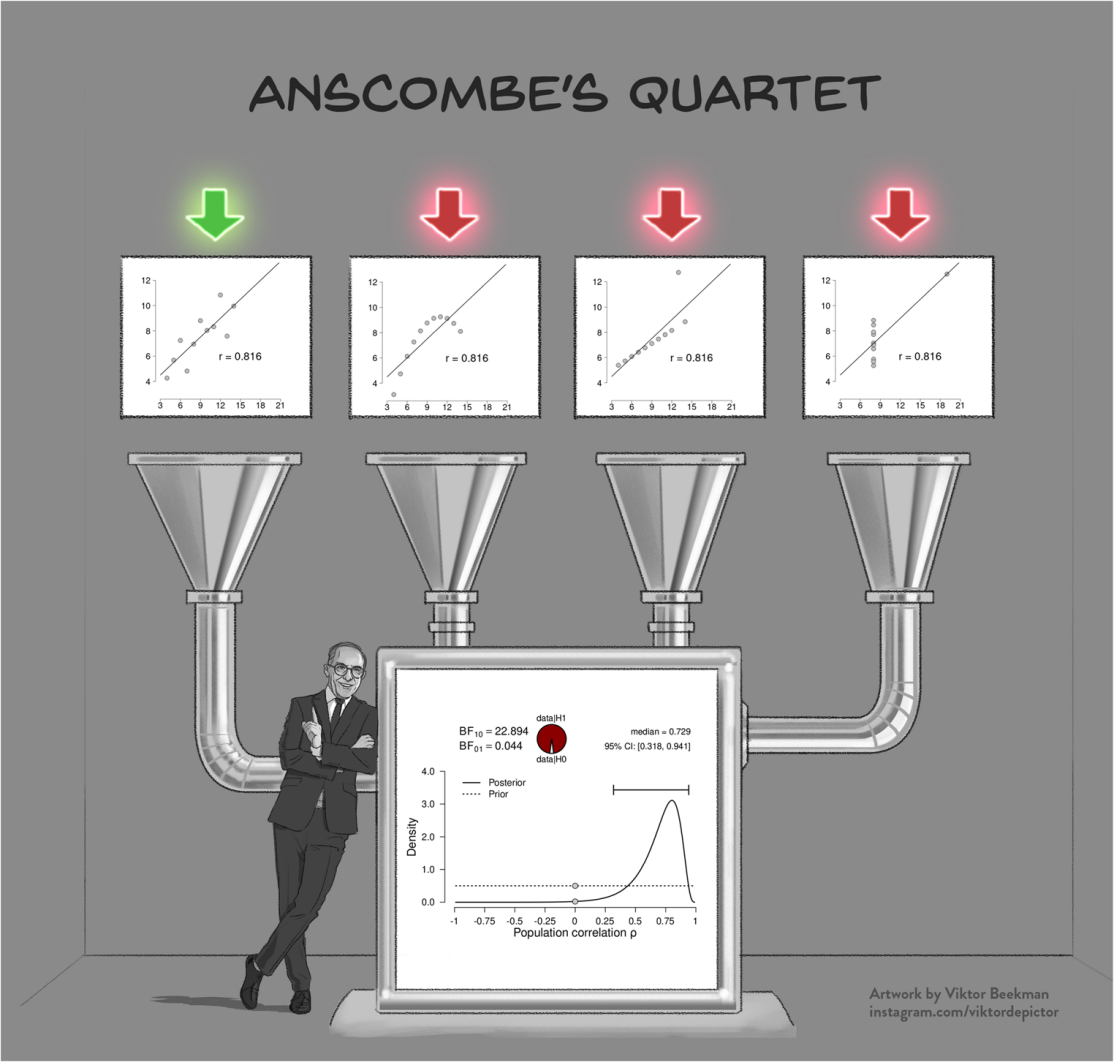
Model misspecification is also a problem for Bayesian analyses. The four scatterplots in the *top panel* show Anscombe’s quartet (Anscombe, 1973); the *bottom panel* shows the corresponding inference, which is identical for all four scatter plots. Except for the leftmost scatterplot, all data violate the assumptions of the linear correlation analysis in important ways

A powerful demonstration of the dangers of failing to check the assumptions is provided by Anscombe’s quartet (Anscombe, 1973; see Fig. 1). The quartet consists of four fictitious data sets of equal size that each have the same observed Pearson’s product moment correlation *r*, and therefore lead to the same inferential result both in a frequentist and a Bayesian framework. However, visual inspection of the scatterplots immediately reveals that three of the four data sets are not suitable for a linear correlation analysis, and the statistical inference for these three data sets is meaningless or even misleading. This example highlights the adage that conducting a Bayesian analysis does not safeguard against general statistical malpractice—the Bayesian framework is as vulnerable to violations of assumptions as its frequentist counterpart. In cases where assumptions are violated, an ordinal or non-parametric test can be used, and the parametric results should be interpreted with caution.

Once the quality of the data has been confirmed, the planned analyses can be carried out. JASP offers a graphical user interface for both frequentist and Bayesian analyses. JASP 0.10.2 features the following Bayesian analyses: the binomial test, the Chi-square test, the multinomial test, the *t* test (one-sample, paired sample, two-sample, Wilcoxon rank-sum, and Wilcoxon signed-rank tests), A/B tests, ANOVA, ANCOVA, repeated measures ANOVA, correlations (Pearson’s *ρ* and Kendall’s *τ*), linear regression, and log-linear regression. After loading the data into JASP, the desired analysis can be conducted by dragging and dropping variables into the appropriate boxes; tick marks can be used to select the desired output.

The resulting output (i.e., figures and tables) can be annotated and saved as a .jasp file. Output can then be shared with peers, with or without the real data in the .jasp file; if the real data are added, reviewers can easily reproduce the analyses, conduct alternative analyses, or insert comments.

### Stereogram example

**Fig. 2:**
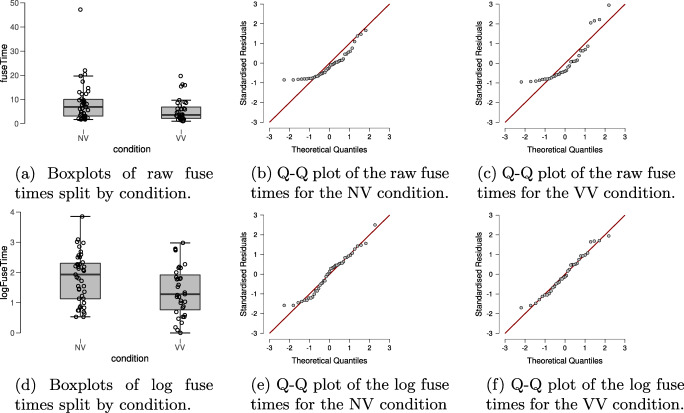
Descriptive plots allow a visual assessment of the assumptions of the *t* test for the stereogram data. The *top row* shows descriptive plots for the raw fuse times, and the *bottom row* shows descriptive plots for the log-transformed fuse times. The *left column* shows boxplots, including the jittered data points, for each of the experimental conditions. The *middle* and *right columns* show parQ-Q plots of the dependent variable, split by experimental condition. Here we see that the log-transformed dependent variable is more appropriate for the *t* test, due to its distribution and absence of outliers. Figures from JASP

In order to check for violations of the assumptions of the *t* test, the top row of Fig. 2 shows boxplots and Q-Q plots of the dependent variable fuse time, split by condition. Visual inspection of the boxplots suggests that the variances of the fuse times may not be equal (observed standard deviations of the NV and VV groups are 8.085 and 4.802, respectively), suggesting the equal variance assumption may be unlikely to hold. There also appear to be a number of potential outliers in both groups. Moreover, the Q-Q plots show that the normality assumption of the *t* test is untenable here. Thus, in line with our analysis plan we will apply the log-transformation to the fuse times. The standard deviations of the log-transformed fuse times in the groups are roughly equal (observed standard deviations are 0.814 and 0.818 in the NV and the VV group, respectively); the Q-Q plots in the bottom row of Fig. 2 also look acceptable for both groups and there are no apparent outliers. However, it seems prudent to assess the robustness of the result by also conducting the Bayesian Mann–Whitney *U* test (van Doorn, Ly, Marsman, & Wagenmakers, 2020) on the fuse times.

**Fig. 3:**
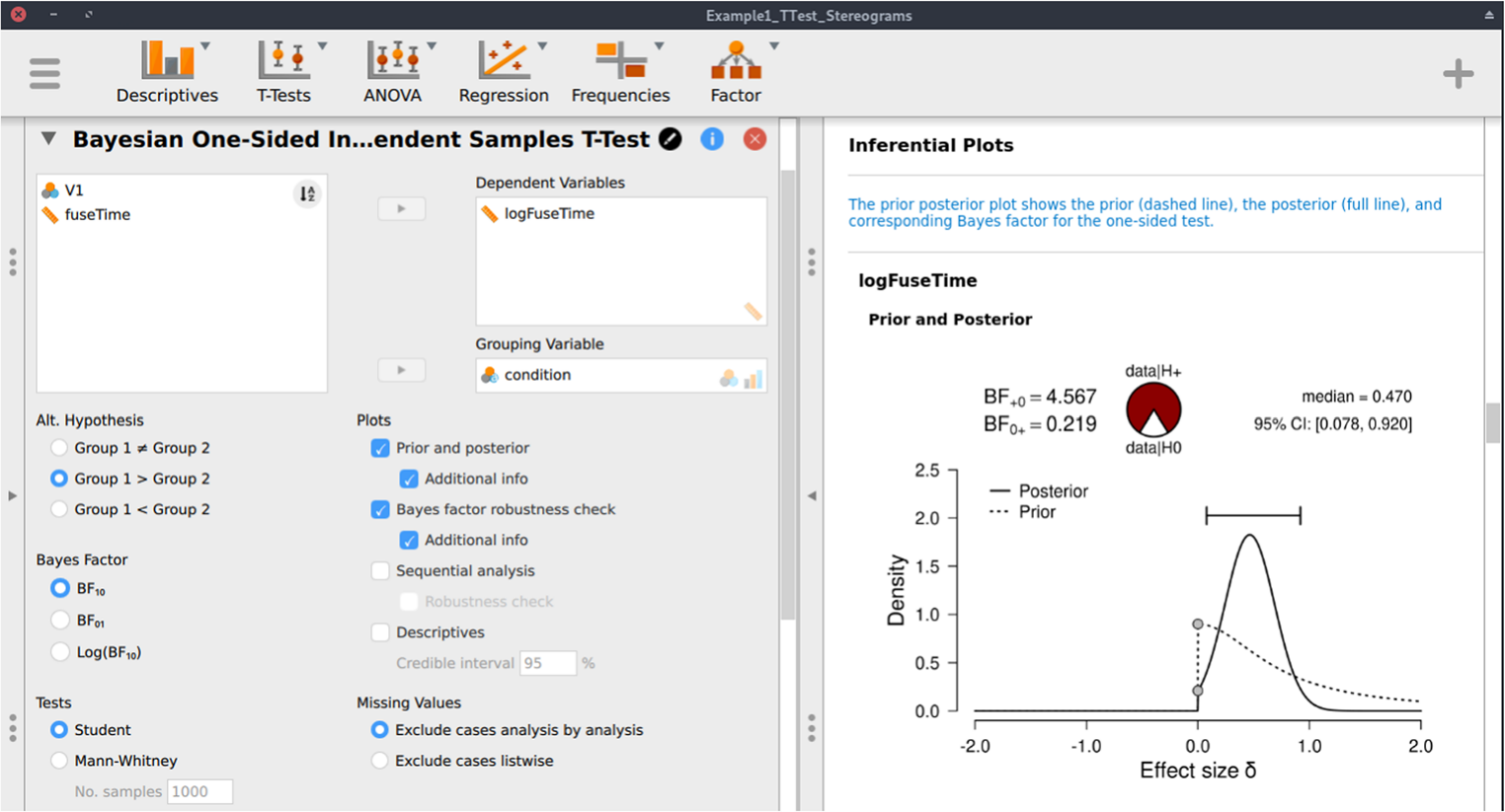
JASP menu for the Bayesian two-sample *t* test. The *left input panel* offers the analysis options, including the specification of the alternative hypothesis and the selection of plots. The *right output panel* shows the corresponding analysis output. The prior and posterior plot is explained in more detail in Fig. 6. The input panel specifies the one-sided analysis for hypothesis testing; a two-sided analysis for estimation can be obtained by selecting “Group 1 ≠ Group 2” under “Alt. Hypothesis”

Following the assumption check, we proceed to execute the analyses in JASP. For hypothesis testing, we obtain a Bayes factor using the one-sided Bayesian two-sample *t* test. Figure 3 shows the JASP user interface for this procedure. For parameter estimation, we obtain a posterior distribution and credible interval, using the two-sided Bayesian two-sample *t* test. The relevant boxes for the various plots were ticked, and an annotated .jasp file was created with all of the relevant analyses: the one-sided Bayes factor hypothesis tests, the robustness check, the posterior distribution from the two-sided analysis, and the one-sided results of the Bayesian Mann–Whitney *U* test. The .jasp file can be found at https://osf.io/nw49j/. The next section outlines how these results are to be interpreted.

## Stage 3: Interpreting the results

With the analysis outcome in hand, we are ready to draw conclusions. We first discuss the scenario of hypothesis testing, where the goal typically is to conclude whether an effect is present or absent. Then, we discuss the scenario of parameter estimation, where the goal is to estimate the size of the population effect, assuming it is present. When both hypothesis testing and estimation procedures have been planned and executed, there is no predetermined order for their interpretation. One may adhere to the adage “only estimate something when there is something to be estimated” (Wagenmakers et al., 2018) and first test whether an effect is present, and then estimate its size (assuming the test provided sufficiently strong evidence against the null), or one may first estimate the magnitude of an effect, and then quantify the degree to which this magnitude warrants a shift in plausibility away from or toward the null hypothesis (but see Box 3).

If the goal of the analysis is hypothesis testing, we recommend using the Bayes factor. As described in Box 1, the Bayes factor quantifies the relative predictive performance of two rival hypotheses (Wagenmakers et al., 2016; see Box 1). Importantly, the Bayes factor is a *relative* metric of the hypotheses’ predictive quality. For instance, if BF_10_ = 5, this means that the data are 5 times more likely under ${\mathscr{H}}_{1}$ than under ${\mathscr{H}}_{0}$. However, a Bayes factor in favor of ${\mathscr{H}}_{1}$ does not mean that ${\mathscr{H}}_{1}$ predicts the data well. As Figure 1 illustrates, ${\mathscr{H}}_{1}$ provides a dreadful account of three out of four data sets, yet is still supported relative to ${\mathscr{H}}_{0}$.

**Fig. 4:**
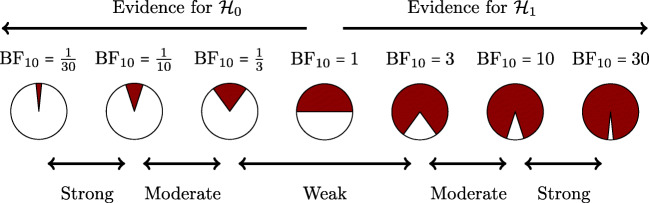
A graphical representation of a Bayes factor classification table. As the Bayes factor deviates from 1, which indicates equal support for ${\mathscr{H}}_{0}$ and ${\mathscr{H}}_{1}$, more support is gained for either ${\mathscr{H}}_{0}$ or ${\mathscr{H}}_{1}$. Bayes factors between 1 and 3 are considered to be weak, Bayes factors between 3 and 10 are considered moderate, and Bayes factors greater than 10 are considered strong evidence. The Bayes factors are also represented as probability wheels, where the ratio of white (i.e., support for ${\mathscr{H}}_{0}$) to red (i.e., support for ${\mathscr{H}}_{1}$) surface is a function of the Bayes factor. The probability wheels further underscore the continuous scale of evidence that Bayes factors represent. These classifications are heuristic and should not be misused as an absolute rule for all-or-nothing conclusions

There can be no hard Bayes factor bound (other than zero and infinity) for accepting or rejecting a hypothesis wholesale, but there have been some attempts to classify the strength of evidence that different Bayes factors provide (e.g., Jeffreys, 1939; Kass & Raftery, 1995). One such classification scheme is shown in Figure 4. Several magnitudes of the Bayes factor are visualized as a probability wheel, where the proportion of red to white is determined by the degree of evidence in favor of ${\mathscr{H}}_{0}$ and ${\mathscr{H}}_{1}$.^4^ In line with Jeffreys, a Bayes factor between 1 and 3 is considered weak evidence, a Bayes factor between 3 and 10 is considered moderate evidence, and a Bayes factor greater than 10 is considered strong evidence. Note that these classifications should only be used as general rules of thumb to facilitate communication and interpretation of evidential strength. Indeed, one of the merits of the Bayes factor is that it offers an assessment of evidence on a continuous scale.

When the goal of the analysis is parameter estimation, the posterior distribution is key (see Box 2). The posterior distribution is often summarized by a location parameter (point estimate) and uncertainty measure (interval estimate). For point estimation, the posterior median (reported by JASP), mean, or mode can be reported, although these do not contain any information about the uncertainty of the estimate. In order to capture the uncertainty of the estimate, an *x%* credible interval can be reported. The credible interval [*L*,*U*] has a *x*% probability that the true parameter lies in the interval that ranges from *L* to *U* (an interpretation that is often wrongly attributed to frequentist confidence intervals, see Morey, Hoekstra, Rouder, Lee, & Wagenmakers, 2016). For example, if we obtain a 95*%* credible interval of [− 1,0.5] for effect size *δ*, we can be 95*%* certain that the true value of *δ* lies between − 1 and 0.5, assuming that the alternative hypothesis we specify is true. In case one does not want to make this assumption, one can present the *unconditional* posterior distribution instead. For more discussion on this point, see Box 3.

### Box 3. Conditional vs. unconditional inference.

**Fig. 5:**
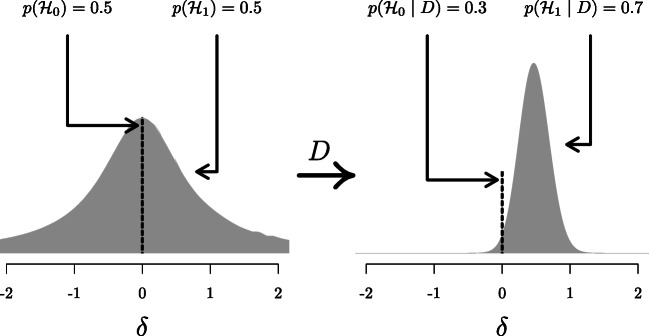
Updating the unconditional prior distribution to the unconditional posterior distribution for the stereogram example. The *left panel* shows the unconditional prior distribution, which is a mixture between the prior distributions under ${\mathscr{H}}_{0}$ and ${\mathscr{H}}_{1}$. The prior distribution under ${\mathscr{H}}_{0}$ is a spike at the null value, indicated by the *dotted line*; the prior distribution under ${\mathscr{H}}_{1}$ is a Cauchy distribution, indicated by the *gray mass*. The mixture proportion is determined by the prior model probabilities $p({\mathscr{H}}_{0})$ and $p({\mathscr{H}}_{1})$. The *right panel* shows the unconditional posterior distribution, after updating the prior distribution with the data *D*. This distribution is a mixture between the posterior distributions under ${\mathscr{H}}_{0}$ and ${\mathscr{H}}_{1}$., where the mixture proportion is determined by the posterior model probabilities $p({\mathscr{H}}_{0} \mid D)$ and $p({\mathscr{H}}_{1} \mid D)$. Since $p({\mathscr{H}}_{1} \mid D) = 0.7$ (i.e., the data provide support for ${\mathscr{H}}_{1}$ over ${\mathscr{H}}_{0}$), about 70% of the unconditional posterior mass is comprised of the posterior mass under ${\mathscr{H}}_{1}$, indicated by the *gray mass*. Thus, the unconditional posterior distribution provides information about plausible values for *δ*, while taking into account the uncertainty of ${\mathscr{H}}_{1}$ being true. In both panels, the *dotted line* and *gray mass* have been rescaled such that the height of the dotted line and the highest point of the gray mass reflect the prior (*left*) and posterior (*right*) model probabilities

A widely accepted view on statistical inference is neatly summarized by Fisher (1925), who states that “it is a useful preliminary before making a statistical estimate $\dots $ to test if there is anything to justify estimation at all” (p. 300; see also Haaf, Ly, & Wagenmakers, 2019). In the Bayesian framework, this stance naturally leads to posterior distributions *conditional* on ${\mathscr{H}}_{1}$, which ignores the possibility that the null value could be true. Generally, when we say “prior distribution” or “posterior distribution” we are following convention and referring to such conditional distributions. However, only presenting conditional posterior distributions can potentially be misleading in cases where the null hypothesis remains relatively plausible after seeing the data. A general benefit of Bayesian analysis is that one can compute an*unconditional* posterior distribution for the parameter using model averaging (e.g., Clyde, Ghosh, & Littman, 2011; Hinne, Gronau, Bergh, & Wagenmakers, 2020). An unconditional posterior distribution for a parameter accounts for both the uncertainty about the parameter within any one model and the uncertainty about the model itself, providing an estimate of the parameter that is a compromise between the candidate models (for more details see Hoeting, Madigan, Raftery, & Volinsky, 1999). In the case of a *t* test, which features only the null and the alternative hypothesis, the unconditional posterior consists of a mixture between a spike under ${\mathscr{H}}_{0}$ and a bell-shaped posterior distribution under ${\mathscr{H}}_{1}$ (Rouder, Haaf, & Vandekerckhove, 2018; van den Bergh, Haaf, Ly, Rouder, & Wagenmakers, 2019). Figure 5 illustrates this approach for the stereogram example.

### Common pitfalls in interpreting Bayesian results

**Fig. 6:**
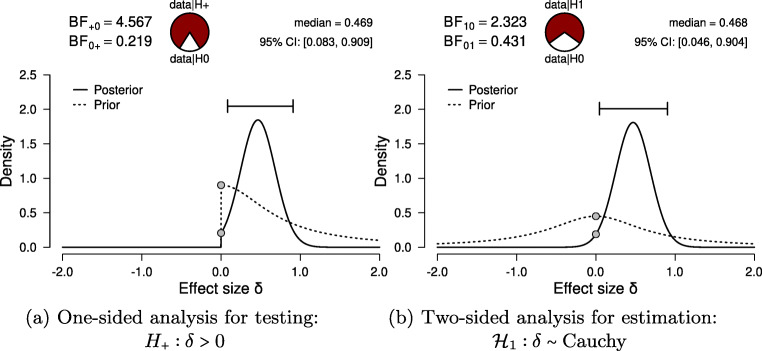
Bayesian two-sample *t* test for the parameter *δ*. The probability wheel on top visualizes the evidence that the data provide for the two rival hypotheses. The two *gray dots* indicate the prior and posterior density at the test value (Dickey & Lientz, 1970; Wagenmakers, Lodewyckx, Kuriyal, & Grasman, 2010). The median and the 95*%* central credible interval of the posterior distribution are shown in the top right corner. The *left panel* shows the one-sided procedure for hypothesis testing and the *right panel* shows the two-sided procedure for parameter estimation. Both figures from JASP

Bayesian veterans sometimes argue that Bayesian concepts are intuitive and easier to grasp than frequentist concepts. However, in our experience there exist persistent misinterpretations of Bayesian results. Here we list five:

- The Bayes factor does not equal the posterior odds; in fact, the posterior odds are equal to the Bayes factor multiplied by the prior odds (see also Equation 1). These prior odds reflect the relative plausibility of the rival hypotheses before seeing the data (e.g., 50/50 when both hypotheses are equally plausible, or 80/20 when one hypothesis is deemed to be four times more plausible than the other). For instance, a proponent and a skeptic may differ greatly in their assessment of the prior plausibility of a hypothesis; their prior odds differ, and, consequently, so will their posterior odds. However, as the Bayes factor is the updating factor from prior odds to posterior odds, proponent and skeptic ought to change their beliefs to the same degree (assuming they agree on the model specification, including the parameter prior distributions).
- Prior model probabilities (i.e., prior odds) and parameter prior distributions play different conceptual roles.^5^ The former concerns prior beliefs about the hypotheses, for instance that both ${\mathscr{H}}_{0}$ and ${\mathscr{H}}_{1}$ are equally plausible a priori. The latter concerns prior beliefs about the model parameters within a model, for instance that all values of Pearson’s *ρ* are equally likely a priori (i.e., a uniform prior distribution on the correlation parameter). Prior model probabilities and parameter prior distributions can be combined to one unconditional prior distribution as described in Box 3 and Fig. 5.
- The Bayes factor and credible interval have different purposes and can yield different conclusions. Specifically, the typical credible interval for an effect size is conditional on ${\mathscr{H}}_{1}$ being true and quantifies the strength of an effect, assuming it is present (but see Box 3); in contrast, the Bayes factor quantifies evidence for the presence or absence of an effect. A common misconception is to conduct a “hypothesis test” by inspecting only credible intervals. Berger (2006, p. 383) remarks: “[...] Bayesians cannot test precise hypotheses using confidence intervals. In classical statistics one frequently sees testing done by forming a confidence region for the parameter, and then rejecting a null value of the parameter if it does not lie in the confidence region. This is simply wrong if done in a Bayesian formulation (and if the null value of the parameter is believable as a hypothesis).”
- The strength of evidence in the data is easy to overstate: a Bayes factor of 3 provides some support for one hypothesis over another, but should not warrant the confident all-or-none acceptance of that hypothesis.
- The results of an analysis always depend on the questions that were asked.^6^ For instance, choosing a one-sided analysis over a two-sided analysis will impact both the Bayes factor and the posterior distribution. For an illustration of this, see Fig. 6 for a comparison between one-sided and a two-sided results.

In order to avoid these and other pitfalls, we recommend that researchers who are doubtful about the correct interpretation of their Bayesian results solicit expert advice (for instance through the JASP forum at http://forum.cogsci.nl).

### Stereogram example

For hypothesis testing, the results of the one-sided *t* test are presented in Fig. 6a. The resulting BF_+ 0_ is 4.567, indicating moderate evidence in favor of ${\mathscr{H}}_{+}$: the data are approximately 4.6 times more likely under ${\mathscr{H}}_{+}$ than under ${\mathscr{H}}_{0}$. To assess the robustness of this result, we also planned a Mann–Whitney *U* test. The resulting BF_+ 0_ is 5.191, qualitatively similar to the Bayes factor from the parametric test. Additionally, we could have specified a multiverse analysis where data exclusion criteria (i.e., exclusion vs. no exclusion), the type of test (i.e., Mann–Whitney *U* vs. *t* test), and data transformations (i.e., log-transformed vs. raw fuse times) are varied. Typically in multiverse analyses these three decisions would be crossed, resulting in at least eight different analyses. However, in our case some of these analyses are implausible or redundant. First, because the Mann–Whitney *U* test is unaffected by the log transformation, the log-transformed and raw fuse times yield the same results. Second, due to the multiple assumption violations, the *t* test model for raw fuse times is misspecified and hence we do not trust the validity of its result. Third, we do not know which observations were excluded by (Frisby & Clatworthy, 1975). Consequently, only two of these eight analyses are relevant.^7^ Furthermore, a more comprehensive multiverse analysis could also consider the Bayes factors from two-sided tests (i.e., BF_10_ = 2.323) for the *t* test and BF_10_ = 2.557 for the Mann–Whitney *U* test). However, these tests are not in line with the theory under consideration, as they answer a different theoretical question (see “Specifying the statistical model” in the Planning section).

For parameter estimation, the results of the two-sided *t* test are presented in Fig. 6a. The 95*%* central credible interval for *δ* is relatively wide, ranging from 0.046 to 0.904: this means that, under the assumption that the effect exists and given the model we specified, we can be 95*%* certain that the true value of *δ* lies between 0.046 to 0.904. In conclusion, there is moderate evidence for the presence of an effect, and large uncertainty about its size.

## Stage 4: Reporting the results

For increased transparency, and to allow a skeptical assessment of the statistical claims, we recommend to present an elaborate analysis report including relevant tables, figures, assumption checks, and background information. The extent to which this needs to be done in the manuscript itself depends on context. Ideally, an annotated .jasp file is created that presents the full results and analysis settings. The resulting file can then be uploaded to the Open Science Framework (OSF; https://osf.io), where it can be viewed by collaborators and peers, even without having JASP installed. Note that the .jasp file retains the settings that were used to create the reported output. Analyses not conducted in JASP should mimic such transparency, for instance through uploading an R-script. In this section, we list several desiderata for reporting, both for hypothesis testing and parameter estimation. What to include in the report depends on the goal of the analysis, regardless of whether the result is conclusive or not.

In all cases, we recommend to provide a complete description of the prior specification (i.e., the type of distribution and its parameter values) and, especially for informed priors, to provide a justification for the choices that were made. When reporting a specific analysis, we advise to refer to the relevant background literature for details. In JASP, the relevant references for specific tests can be copied from the drop-down menus in the results panel.

**Fig. 7:**
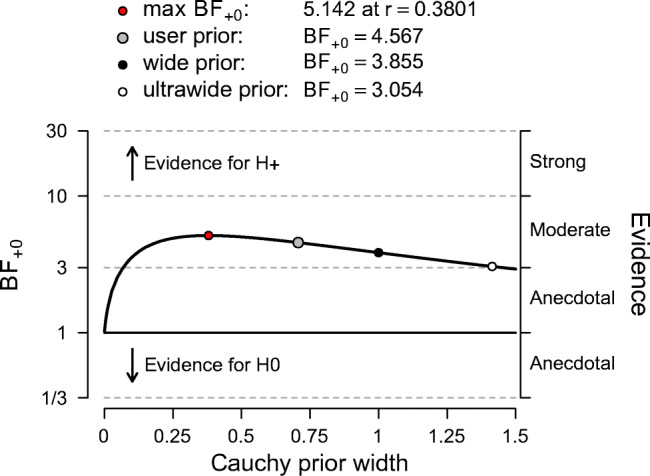
The Bayes factor robustness plot. The maximum BF_+ 0_ is attained when setting the prior width *r* to 0.38. The plot indicates BF_+ 0_ for the user specified prior ($r = {1}/{\sqrt {2}}$), wide prior (*r* = 1), and ultrawide prior ($r = \sqrt {2}$). The evidence for the alternative hypothesis is relatively stable across a wide range of prior distributions, suggesting that the analysis is robust. However, the evidence in favor of ${\mathscr{H}}_{+}$ is not particularly strong and will not convince a skeptic

When the goal of the analysis is hypothesis testing, it is key to outline which hypotheses are compared by clearly stating each hypothesis and including the corresponding subscript in the Bayes factor notation. Furthermore, we recommend to include, if available, the Bayes factor robustness check discussed in the section on planning (see Fig. 7 for an example). This check provides an assessment of the robustness of the Bayes factor under different prior specifications: if the qualitative conclusions do not change across a range of different plausible prior distributions, this indicates that the analysis is relatively robust. If this plot is unavailable, the robustness of the Bayes factor can be checked manually by specifying several different prior distributions (see the mixed ANOVA analysis in the online appendix at https://osf.io/wae57/ for an example). When data come in sequentially, it may also be of interest to examine the sequential Bayes factor plot, which shows the evidential flow as a function of increasing sample size.

When the goal of the analysis is parameter estimation, it is important to present a plot of the posterior distribution, or report a summary, for instance through the median and a 95*%* credible interval. Ideally, the results of the analysis are reported both graphically and numerically. This means that, when possible, a plot is presented that includes the posterior distribution, prior distribution, Bayes factor, 95*%* credible interval, and posterior median.^8^

Numeric results can be presented either in a table or in the main text. If relevant, we recommend to report the results from both estimation and hypothesis test. For some analyses, the results are based on a numerical algorithm, such as Markov chain Monte Carlo (MCMC), which yields an error percentage. If applicable and available, the error percentage ought to be reported too, to indicate the numeric robustness of the result. Lower values of the error percentage indicate greater numerical stability of the result.^9^ In order to increase numerical stability, JASP includes an option to increase the number of samples for MCMC sampling when applicable.

### Stereogram example

This is an example report of the stereograms *t* test example:

Here we summarize the results of the Bayesian analysis for the stereogram data. For this analysis we used the Bayesian *t* test framework proposed by (see also; Jeffreys, 1961; Rouder et al., 2009). We analyzed the data with JASP (JASP Team, 2019). An annotated .jasp file, including distribution plots, data, and input options, is available at https://osf.io/25ekj/. Due to model misspecification (i.e., non-normality, presence of outliers, and unequal variances), we applied a log-transformation to the fuse times. This remedied the misspecification. To assess the robustness of the results, we also applied a Mann–Whitney *U* test.

First, we discuss the results for hypothesis testing. The null hypothesis postulates that there is no difference in log fuse time between the groups and therefore ${\mathscr{H}}_{0}: \delta = 0$. The one-sided alternative hypothesis states that only positive values of *δ* are possible, and assigns more prior mass to values closer to 0 than extreme values. Specifically, *δ* was assigned a Cauchy prior distribution with $r ={1}/{\sqrt {2}}$, truncated to allow only positive effect size values. Figure 6a shows that the Bayes factor indicates evidence for ${\mathscr{H}}_{+}$; specifically, BF_+ 0_ = 4.567, which means that the data are approximately 4.5 times more likely to occur under ${\mathscr{H}}_{+}$ than under ${\mathscr{H}}_{0}$. This result indicates moderate evidence in favor of ${\mathscr{H}}_{+}$. The error percentage is < 0.001*%*, which indicates great stability of the numerical algorithm that was used to obtain the result. The Mann–Whitney *U* test yielded a qualitatively similar result, BF_+ 0_ is 5.191. In order to assess the robustness of the Bayes factor to our prior specification, Fig. 7 shows BF_+ 0_ as a function of the prior width *r*. Across a wide range of widths, the Bayes factor appears to be relatively stable, ranging from about 3 to 5.

Second, we discuss the results for parameter estimation. Of interest is the posterior distribution of the standardized effect size *δ* (i.e., the population version of Cohen’s *d*, the standardized difference in mean fuse times). For parameter estimation, *δ* was assigned a Cauchy prior distribution with $r ={1}/{\sqrt {2}}$. Figure 6b shows that the median of the resulting posterior distribution for *δ* equals 0.47 with a central 95% credible interval for *δ* that ranges from 0.046 to 0.904. If the effect is assumed to exist, there remains substantial uncertainty about its size, with values close to 0 having the same posterior density as values close to 1.

## Limitations and challenges

The Bayesian toolkit for the empirical social scientist still has some limitations to overcome. First, for some frequentist analyses, the Bayesian counterpart has not yet been developed or implemented in JASP. Secondly, some analyses in JASP currently provide only a Bayes factor, and not a visual representation of the posterior distributions, for instance due to the multidimensional parameter space of the model. Thirdly, some analyses in JASP are only available with a relatively limited set of prior distributions. However, these are not principled limitations and the software is actively being developed to overcome these limitations. When dealing with more complex models that go beyond the staple analyses such as *t* tests, there exist a number of software packages that allow custom coding, such as JAGS (Plummer, 2003) or Stan (Carpenter et al., 2017). Another option for Bayesian inference is to code the analyses in a programming language such as R (R Core Team, 2018) or Python (van Rossum, 1995). This requires a certain degree of programming ability, but grants the user more flexibility. Popular packages for conducting Bayesian analyses in R are the BayesFactor package (Morey & Rouder, 2015) and the brms package (Bürkner, 2017), among others (see https://cran.r-project.org/web/views/Bayesian.html for a more exhaustive list). For Python, a popular package for Bayesian analyses is PyMC3 (Salvatier, Wiecki, & Fonnesbeck, 2016). The practical guidelines provided in this paper can largely be generalized to the application of these software programs.

## Concluding comments

We have attempted to provide concise recommendations for planning, executing, interpreting, and reporting Bayesian analyses. These recommendations are summarized in Table 1. Our guidelines focused on the standard analyses that are currently featured in JASP. When going beyond these analyses, some of the discussed guidelines will be easier to implement than others. However, the general process of transparent, comprehensive, and careful statistical reporting extends to all Bayesian procedures and indeed to statistical analyses across the board.

## Footnotes

1 The variables are participant number, the time (in seconds) each participant needed to see the hidden figure (i.e., fuse time), experimental condition (VV = with visual information, NV = without visual information), and the log-transformed fuse time.

2 A one-sided alternative hypothesis makes a more risky prediction than a two-sided hypothesis. Consequently, if the data are in line with the one-sided prediction, the one-sided alternative hypothesis is rewarded with a greater gain in plausibility compared to the two-sided alternative hypothesis; if the data oppose the one-sided prediction, the one-sided alternative hypothesis is penalized with a greater loss in plausibility compared to the two-sided alternative hypothesis.

3 The fat-tailed Cauchy distribution is a popular default choice because it fulfills particular desiderata, see (Jeffreys, 1961;Liang, German, Clyde, & Berger, 2008; Ly et al., 2016; Rouder, Speckman, Sun, Morey, & Iverson, 2009) for details.

4 Specifically, the proportion of red is the posterior probability of ${\mathscr{H}}_{1}$ under a prior probability of 0.5; for a more detailed explanation and a cartoon see https://tinyurl.com/ydhfndxa

5 This confusion does not arise for the rarely reported unconditional distributions (see Box 3).

6 This is known as Jeffreys’s platitude: “The most beneficial result that I can hope for as a consequence of this work is that more attention will be paid to the precise statement of the alternatives involved in the questions asked. It is sometimes considered a paradox that the answer depends not only on the observations but on the question; it should be a platitude” (Jeffreys, 1939, p.vi).

7 The Bayesian Mann–Whitney *U* test results and the results for the raw fuse times are in the .jasp file at https://osf.io/nw49j/.

8 The posterior median is popular because it is robust to skewed distributions and invariant under smooth transformations of parameters, although other measures of central tendency, such as the mode or the mean, are also in common use.

9 We generally recommend error percentages below 20% as acceptable. A 20% change in the Bayes factor will result in one making the same qualitative conclusions. However, this threshold naturally increases with the magnitude of the Bayes factor. For instance, a Bayes factor of 10 with a 50% error percentage could be expected to fluctuate between 5 and 15 upon recomputation. This could be considered a large change. However, with a Bayes factor of 1000 a 50% reduction would still leave us with overwhelming evidence.
